# Reviewing the History of Pandemic Influenza: Understanding Patterns of Emergence and Transmission

**DOI:** 10.3390/pathogens5040066

**Published:** 2016-12-06

**Authors:** Patrick R. Saunders-Hastings, Daniel Krewski

**Affiliations:** McLaughlin Centre for Population Health Risk Assessment, University of Ottawa, 850 Peter Morand Crescent, Ottawa, ON K1G 5Z3, Canada; dkrewski@uottawa.ca

**Keywords:** pandemic, influenza, globalization, intervention

## Abstract

For centuries, novel strains of influenza have emerged to produce human pandemics, causing widespread illness, death, and disruption. There have been four influenza pandemics in the past hundred years. During this time, globalization processes, alongside advances in medicine and epidemiology, have altered the way these pandemics are experienced. Drawing on international case studies, this paper provides a review of the impact of past influenza pandemics, while examining the evolution of our understanding of, and response to, these viruses. This review argues that pandemic influenza is in part a consequence of human development, and highlights the importance of considering outbreaks within the context of shifting global landscapes. While progress in infectious disease prevention, control, and treatment has improved our ability to respond to such outbreaks, globalization processes relating to human behaviour, demographics, and mobility have increased the threat of pandemic emergence and accelerated global disease transmission. Preparedness planning must continue to evolve to keep pace with this heightened risk. Herein, we look to the past for insights on the pandemic experience, underlining both progress and persisting challenges. However, given the uncertain timing and severity of future pandemics, we emphasize the need for flexible policies capable of responding to change as such emergencies develop.

## 1. Introduction

Influenza pandemics, distinguished from epidemics on the basis of their geographical spread, have caused significant illness, death, and disruption for centuries. In recent decades, however, globalization has driven social and economic changes that have enhanced the threat of disease emergence and accelerated the spread of novel viruses. Conversely, globalization has also facilitated international cooperation, promoting advances in disease research and surveillance. Collectively, these changes alter the way that pandemics originate and are experienced, understood, and controlled. This paper examines how changes in human demographics, economic systems, medical capabilities, and epidemiological practices have affected the consequences of pandemic influenza, and the approaches to, and challenges in, responding to outbreaks. In the past century, four novel influenza strains have emerged, each leading to a global pandemic. These pandemics are addressed in sequence, highlighting pandemic origins, responses, and the burdens of the consequences. This review is complemented by an analysis of how change in the intervening years between pandemics, with respect to globalization processes and to infectious disease practices, impacted exposure to, and preparedness for, the subsequent pandemic. We begin with a brief discussion of the disease and a background on pandemics from the more distant past.

## 2. The Disease

Influenza viruses are named for the Latin *Influentia*, or “influence” [1]. Belonging to the Orthomyxoviridae family, they have a genome made up of eight segments, which together code for ten proteins [2]. Strains can be separated into types A, B, and C. The present review deals exclusively with influenza type A. While types A and B are responsible for the majority of morbidity and mortality, type A is the only one with pandemic potential [3]. This is because type A is the only strain with an animal reservoir. Aquatic birds and swine are important reservoirs for influenza A, presenting obstacles to eradication and providing opportunities for viral mutation and reassortment [4].

The highly variable nature of influenza genetic material prevents maintenance of an adequate immune response acquired from previous infections, leading to annual epidemics of “seasonal influenza” [5]. Associated lower respiratory infections cause between 28,000 and 111,500 deaths globally in children under five years of age alone [6]. While the focus of this work is on the more catastrophic potential of pandemic strains, the continued burden of seasonal influenza strains suggests that co-benefits of pandemic flu planning extend to seasonal flu preparedness, as well as to other infectious disease outbreaks. 

Influenza virus subtypes are distinguished based upon the antigenic properties of two surface glycoproteins: hemagglutinin (H) and neuraminidase (N), which promote and coordinate host cell entry and exit, respectively. The United States Centers for Disease Control and Prevention identifies 18 H subtypes and 11 N subtypes, for a theoretical total of 198 strain variations [7]. However, only H1, H2, and H3 are known to have achieved substantial human-to-human transmission [8].

Antigenic *drift*, a result of mutations in the genes encoding the H and N antigens, occurs continuously and drives the evasion of host immune system response that produces outbreaks of seasonal influenza [9]. This is the result of the RNA stranded genome, which lacks the ability to correct mistakes during replication. Antigenic *shift* is far rarer, and arises from the viral reassortment of two different influenza viruses that co-infect the same host, creating a new virus [9]. If the novel virus has the ability to infect humans and achieve human-to-human transmission, and possesses virulence for humans, a pandemic may arise, as humans are unlikely to have appreciable immunity to the novel strain. Within the past one hundred years, four pandemics have resulted from the emergence of a novel influenza strain for which humans possessed little or no immunity: the H1N1 Spanish flu (1918), the H2N2 Asian flu (1957), the H3N2 Hong Kong flu (1968), and the H1N1 swine flu (2009). These are summarized in Table 1. Most of our understanding of pandemics has arisen in this period, and is the primary focus of this article; however, a brief discussion of more ancient pandemics is included for historical context.

## 3. From Ancient Times to the Machine Age

Influenza epidemics and pandemics have been occurring for centuries. Epidemics result in local spikes in infection incidence, and tend to be driven by seasonal influenza strains, whereas pandemics are epidemics that spread globally. Greek writings from 412 BC describe what medical historians believe may have been an influenza outbreak [10,11]. However, because the virus was not isolated and identified until the twentieth century, medical historians are restricted to searching for known signs and symptoms of influenza infection. An overall lack of data up until 1500 precludes significant analysis of the influenza outbreaks of the more distant past [12].

There is general agreement that an outbreak in 1580 represents the first influenza pandemic [12,13]. It is likely that the strain emerged that summer in Asia, spreading by land routes to Asia Minor and North Africa before moving across Europe and into North America [14]. Disruption, illness, and death were widely reported [15]. The first reference to “influenza” in scientific literature appeared in 1650 [12]. From this date, the history of pandemics is more reliably documented. 

The first pandemic of the eighteenth century began in the spring of 1729 in Russia, spreading across Europe during the ensuing six months and around the globe over the next three years [14,16,17,18]. As with more recent pandemics, the outbreak occurred in multiple waves, with higher associated morbidity and mortality in later stages [15,17]. The second pandemic of that century appears to have begun in China in the autumn of 1781 [14,18]. It spread through Russia and Europe over a period of eight months, with a particularly high attack rate among young adults [19]. 

The major pandemic of the nineteenth century began in the winter of 1830 in China [12]. Reported to be of similar severity to the 1918 Spanish flu pandemic, it spread across Southeast Asia, Russia, and Europe, and into North America by 1831 [14,15,17]. Despite a high illness attack rate, associated mortality was low [12]. In the winter of 1889, another pandemic emerged in Russia, spreading by rail and sea across Europe and North America [14,17]. With an estimated case fatality rate in the range of 0.1%–0.28%, the outbreak killed about one million people globally [20]. This pandemic spread at a faster rate than previous ones, and may provide the first indication of the accelerated spread of emergent diseases as a result of progress in transportation technology.

There remains considerable uncertainty over when and where influenza pandemics have emerged in the past four hundred years [12,13]. It is worth noting, however, that in each of the ten pandemics where data on emergence is available, either China, Russia, or more broadly Asia have been identified as the likely point of origin [12]. Scholars tend to give a fairly consistent estimated interval of 10–50 years between influenza pandemics [10,12,13]. This is a very broad window, suggesting that pandemics occur with an irregularity that prevents accurate prediction of emergence. 

Several patterns emerge in examining early pandemics. The first is an overall lack of quality, rigour, and validity in the available evidence. Inconsistencies in disease recognition and reporting make it difficult to estimate with certainty the attributable health burden of these disease outbreaks. The second pattern is the relationship between the rate of disease spread and the transportation revolution of the eighteenth and nineteenth centuries. Influenza outbreaks in an area tend to last between six and ten weeks, and were previously contained to spreading along commercial trading routes, whether by foot, horse, or downstream by boat. However, the industrial revolution led to the expansion of road systems and the advent of the steam engine, along with the promotion of steamboats and railroads for trade and travel. These technological advances led to major increases in human mobility, both within and across countries, and quickly became the primary vector of disease spread throughout the world.

Public health practice in the time of these pandemics was still rudimentary, and knowledge of disease prevention and management was poor. Vaccinations, antivirals, and antibiotics to treat secondary infections had yet to be developed, and systematic response plans are not evident. Progress in both public health practice and infectious disease management would quickly become a priority, driven in part by the devastation wrought by the 1918 Spanish flu pandemic.

## 4. 1918–1920: Spanish Flu

In 1918, an H1N1 influenza strain emerged to cause the Spanish flu pandemic, a disaster that has been called the ‘greatest medical holocaust in history’ [21]. While the timing and number of waves was not consistent globally, the pandemic is generally viewed to have had three distinct waves: the spring of 1918, the fall of 1918, and the winter of 1918–1919 [22,23]. While the first and third waves were fairly mild, the second wave resulted in catastrophic global losses, with deaths reaching into the tens of millions. Global death toll estimates have been revised upwards in the decades since the pandemic. Initial assessments in the 1920s estimated deaths at around 21.5 million; this was subject to a recalculation in 1991 to between 24.7 and 39.3 million [24,25]. A 2002 estimate put deaths at around fifty million, with an upper limit as high as a hundred million deaths [23]. More recent estimates tend to fall in this broad range, with 40–50 million deaths being most commonly reported [2,13,22,26,27]. Most of these deaths occurred over a four-month period in the autumn of 1918 [28]. 

Despite poor or absent data for many countries, the virus is believed to have infected over half of the world’s population at the time [12]. Social and economic disruption were also prevalent, as absenteeism led to shutdowns of schools and businesses (many of which went bankrupt) [29]. Claims against life insurance policies soared by as much as 745% [29]. The pandemic strain differed from seasonal epidemics in terms of its disproportionate burden among young people, particularly previously healthy individuals between 18 and 40 years of age [13,22,23]. While the reasons for this are poorly understood, one possible explanation relates to the role of infection in turning the immune system against itself, triggering a dangerous and potentially deadly cytokine storm [27]. Consequently, those with the most robust immune systems may have been at greatest risk. This shift in mortality risk towards younger age groups may also have been due to prior exposure to a genetically similar strain among older age groups [30]. 

More specific accounts of the experience of particular regions are broadly available, including for North America [14,24], Africa [31], Europe [32], and Australia [33]. However, many of these accounts have not been subjected to peer-review, and reports, observations, and conclusions often rely on assumptions and estimates made necessary by limited or unreliable data. This tends to be the greatest challenge associated with analyzing pandemics that occurred before the advent of systematic disease surveillance and reporting. Nevertheless, while the broad estimated mortality range highlights problems with data collection, missing records, and misdiagnosis, it still provides a sense of the severity of the pandemic.

The origin of Spanish flu has been subject to uncertainty and debate, with proposed origins tending to center on the United States and China [12,28,29]. Recently, Humphries reviewed evidence of early outbreaks of unidentified, virulent respiratory diseases shortly before the emergence of the 1918 flu [22]. Despite limited availability of epidemiological evidence to confirm the theory, he presents data from several countries which suggests that the origin of the first wave of Spanish flu was an outbreak in China that was misidentified as pneumonic plague. It then would have spread across the globe through the Chinese Labour Corps (CLC), a group that, from 1916 to the end of the First World War, sent over 100,000 laborers to Europe to support the Allied war effort [34]. Originally reaching Europe via checkpoints in Singapore, Durban, Cape Town, or North Africa, workers were later redirected to Vancouver, to be brought by train to Halifax and sent across the Atlantic [22]. In April 1918, a transport of workers experienced an outbreak of supposed plague; shortly after, the program was cancelled for fear of transporting infected workers. However, during this time, there was a spike in influenza cases reported among Canadian soldiers assigned to guard and transport the Chinese workers [22]. The argument for a Chinese origin is supported by observations of a (relatively) milder second wave in both China and Chinese populations in Canada, suggesting they may have been subject to previous exposure to the virus [22,35]. Alternatively, Cheng and Leung argue that China’s escape from a more severe pandemic was due to the role of traditional Chinese herbal medicine [36]. 

The origin of the second wave of Spanish influenza, which would go on to produce the majority of infections and deaths associated with the pandemic, is more generally agreed upon. It apparently emerged in Southern England, specifically in Plymouth and Devonport [22]. From the harbour town of Plymouth—a popular arrival site for CLC workers—the merchant ship *Mantua* transported the virus to Freetown, Sierra Leone [37]. Also a popular harbour town, the Freetown outbreak would spread the second wave of influenza across Africa, as ships would make port to refuel before traveling further South [22]. Meanwhile, New Zealand soldiers sailing to and from the War in Europe were also infected when they stopped in Freetown on their way; over 8500 New Zealanders would die from influenza and pneumonia in just six weeks [37]. Around this time, a boat from Plymouth landed in Boston, seeding the infection in North America. Over the following four months, as many as 675,000 Americans, 300,000 Mexicans, and 50,000 Canadians would die from infection [23,29]. 

In terms of overall illness and death, the Spanish flu pandemic is among the greatest public health disasters in recorded history [21]. It was the result of a highly pathogenic, transmissible strain of influenza that emerged in a time when populations that previously would have had limited contact with one another were brought together by World War I (WWI). While previous pandemics travelled mostly along trade routes and communication lines, the spread of the 1918 outbreak was accelerated by the military context in which it developed. Meanwhile, trench warfare in Europe provided ideal conditions—poor sanitation, overcrowding, and limited health services—to facilitate disease transmission [22]. 

At the same time, these problems were compounded by a public health system that was unprepared for an influenza strain of such pandemic potential. In 1918, experts still believed that influenza was caused by the *Bacillus influenzae* bacterium, though doubts were raised when physicians were unable to find any bacilli during autopsies [38]. Doctors also had a difficult time diagnosing influenza infection, often mistaking it for the common cold, cholera, or bubonic plague [29]. They did recognize, however, that the routes of influenza transmission were via infectious respiratory droplets from the nose and throat. Further, though at this time some countries did have regulations for reportable diseases, influenza was not one of them [28]. As such, early stages of local outbreaks tended to be largely ignored, and action was often not taken until it was too late to achieve containment. Influenza would become a reportable disease early in the second wave of the pandemic, but not until infection was so widespread as to preclude accurate record keeping [29].

In 1918, effective vaccines and antibiotics to prevent influenza and treat secondary bacterial pneumonia were still decades away. Meanwhile, medical personnel were in short supply due to WWI. Hospital beds were also limited, and community centers and local schools were repurposed as surge centers. People who fell ill would turn to a range of ineffective drugstore and home remedies, such as topical creams or a mixture of water, salt, and coal oil [29]. Some doctors even prescribed alcohol consumption as a means of infection prevention; this produced little more than a surge in liquor demand [29]. 

Efforts to control outbreaks had to rely on non-pharmaceutical interventions (NPIs), such as quarantines, school closure, banning public gatherings, and infection prevention practices like cough and sneeze etiquette and use of facemasks [39]. Interventions were of variable effectiveness. For example, the gauze facemasks would have been effective in preventing bacterial infection, but were too porous to stop viral penetration, and many resisted their use regardless. Governments issued directives on the dangers of influenza, but these were often poorly understood or ignored [29]. A recent analysis of NPI strategies implemented in the United States during the second and third wave in 1918, however, found that they were significantly associated with reductions in mortality and delays in reaching pandemic peaks [39]. The most effective strategies combined different types of NPI in a layered approach, with a combination of school closure and banning public gatherings being found to be the most successful [39]. Interventions implemented early and for longer duration also had a greater impact. NPIs alone are unlikely to prevent or contain a pandemic, as they do not affect susceptibility to, or infectivity from, viral infection. It is for this reason that, if NPIs are discontinued prematurely, infection may quickly return to its normal transmission patterns, leaving the ultimate impact of the outbreak unchanged. However, if implemented properly, NPIs have the potential to delay and flatten pandemic peaks in a way that reduces mortality and alleviates stress on the health care system. 

Spanish flu brought illness, death, and loss to tens of millions of people around the globe and is the worst pandemic in recorded history. However, it also brought a sense of urgency to improve public health, which led to advances in medical sciences, public health planning, and international cooperation. It would be almost 40 years before another global influenza pandemic and, when it arrived, countries found themselves much better prepared.

## 5. 1920s–1950s: Discoveries, Vaccines, and the WHO

In the years following the Spanish flu outbreak, the H1N1 virus continued to circulate, though it did not re-emerge to cause illness and death on a similar scale. In the decades before another pandemic strain emerged, global and public health would advance by leaps and bounds. With respect to pandemic influenza, three areas of progress may be highlighted: virus isolation and identification, the development of vaccines, and the advance of global health diplomacy.

Richard Shope first isolated the influenza virus in the laboratory in 1931, extracting it from infected pigs [40]. Not long after, Smith, Andrewes, and Laidlaw isolated the virus in humans, disproving the widely held belief that influenza was a bacterial infection [41]. This was a significant breakthrough for efforts towards diagnosis, surveillance, and vaccine development. The first vaccine for the influenza virus was developed in parallel by multiple researchers during the late 1930s and early 1940s, with credit most often going to Jonas Salk and Thomas Francis [42,43]. Vaccines in this period were not as safe as modern vaccines, and impurities would sometimes cause flu-like symptoms such as fever, aches, and fatigue [2]. Meanwhile, poor surveillance capabilities made it difficult to properly match the vaccine with the circulating influenza strain. In 1947, for example, an epidemic emerged when antigenic drift resulted in changes to the hemagglutinin antigen such that the flu vaccine did not provide any protection against influenza [44]. Fortunately, the strain was not very severe, and a pandemic did not occur [45]. The discovery and isolation of the influenza virus would dramatically change the way societies approached flu prevention and control. Meanwhile, the discovery of penicillin in 1929 would provide health planners with an important tool for treating secondary bacterial pneumonia, the primary cause of death during influenza pandemics [46]. In addition, positive-pressure ventilators for use in intensive care units (ICUs) were developed in the 1940s; this would also improve health outcomes in complicated cases [47]. Taken together, these advances have helped prevent another pandemic with a case fatality rate similar to that of the Spanish flu. 

During the Spanish flu pandemic, there was little meaningful coordination among jurisdictions. There were several reasons for this. First, meaningful international cooperation for the control of infectious diseases was still in its infancy. Beginning in 1851, a series of International Sanitary Conferences began to bring countries together to address infectious disease control; however, the early treaties arising from these conferences, with their focus on sanitation, proved of limited use during an influenza pandemic [48]. Meanwhile, international organizations with mandates to coordinate and inform infectious disease response were inadequate. International bodies such as the Pan American Sanitary Bureau (which would later become the Pan American Health Organization) and the Office International d’Hygiène Publique (in Paris, France), were founded in the early twentieth century, but did not have the size, range, or expertise to effectively contribute to the Spanish flu response [48]. Meanwhile, the League of Nations, arguably the first global political system, was founded in 1919, establishing a health organization in 1923 (replaced by the World Health Organization in 1948) [48,49]. These international bodies would play an important role in later pandemics. Further, many national health institutions did not yet exist, and provincial/state health departments were small [50]. In Canada, largely as a result of the disorganized response to Spanish flu, legislation for the establishment of a federal health department was introduced in March 1919 [50]. In the United States, the Communicable Disease Center (now the Centers for Disease Control and Prevention) was not formed until 1946 [51]. The result was that states planned and implemented very different control strategies, often with little insight from the experience and best practices of other states [39]. In the absence of these national and international coordinating bodies, a lack of communication and reporting between jurisdictions impeded more effective responses. The size and responsibilities of local, state, provincial, and federal health departments expanded over the upcoming decades.

In the inter-pandemic period between 1918 and 1957, the world experienced massive growth in population, trade, and travel. In 1918, the global population was around 1.8 billion; by 1957 that figure had risen to 2.8 billion [52,53]. Meanwhile, international travel for both business and leisure had been increasing steadily for years. However, with the arrival of commercial jet aircraft in the 1950s, the number of international travellers began to climb rapidly [54]. A similar trend is apparent in international trade, which has grown 140-fold from the industrial revolution of the nineteenth century to the twenty-first century [55]. Though the globalization of trade stalled from 1914 to 1945—limited by WWI, the Great Depression, and WWII—it, too, would re-emerge in the 1950s, initiating what has been called the “second age of globalization” (the explosion of trade, capital, and migration during the industrial revolution is considered the first) [55]. The beginning of this second age may be traced to the 1944–1947 establishment of the United Nations and three multilateral economic institutions known collectively as the Bretton Woods system: the World Bank, the International Monetary Fund, and the General Agreement on Tariffs and Trade [55]. These organizations set the stage for unprecedented international trade cooperation and liberalization, catalyzing the formation of multinational institutions and the international movement of goods, services, and information on a scale wholly different from that seen before the First World War. Therefore, while three decades of advances in medical sciences, public health practice, and international political cooperation may have improved preparedness for pandemic influenza emergence, three decades of population growth and the globalization of trade and travel increased the risk of disease emergence and spread. This contributed to the emergence of two global, albeit mild, influenza pandemics within a decade of each other.

## 6. 1957–1958: Asian Flu

After almost forty years of H1N1 being the only influenza strain in circulation, a new strain emerged to cause another influenza pandemic. In February 1957, a new influenza strain was detected in the Yunnan Province of China [14]. Humans under 65 possessed no immunity to this H2N2 strain, suggesting prior viral circulation and exposure at some point in the late nineteenth century [56]. The virus spread to Hong Kong in April, then to Singapore, Taiwan, and Japan before spreading globally throughout the summer of 1957 [56]. By June, it was reported in twenty countries [57]. In the United States, it was first reported on naval bases, again suggesting the role of military routes in global disease diffusion [51]. The pandemic was spread primarily via land and sea routes, with air travel playing only a small part in disease spread [14]. The majority of global transmission occurred over land routes from Russia to Scandinavia and Eastern Europe and at an international conference held in Iowa [58,59]. As with the 1918 Spanish flu, Asian flu would reappear in successive, unpredictable waves, with the second wave being more severe than the first [60]. Although the virus was circulating during the summer, community-wide transmission was limited; it was not until schools re-opened in the fall that broader transmission was triggered, with clinical attack rates in schools of 40%–60% [61].

The strain came to be known as the Asian flu, and was a comparatively mild influenza pandemic. With a maximum case fatality rate estimated to be about 0.67%, it caused between one and two million deaths worldwide [26,60,61,62]. A recent study by Viboud and colleagues used historical mortality data from 39 countries to estimate an average pandemic-associated excess respiratory mortality rate of 1.9 per 10,000 people [62]. Excess pandemic-related mortality between 1957 and 1959 in the United States was estimated to be 0.83/10,000, compared to 1.79/10,000 over the same period in Canada. As with Spanish flu, the mortality curve shifted towards younger age groups, a distinguishing characteristic of influenza pandemics [63]. Younger age groups dominated in terms of attack rates, suggesting pre-existing immunity in older groups [64].

There was some societal disruption due to school and workplace absenteeism, though this was mostly concentrated among children, school teachers, and healthcare workers [65]. During the pandemic peak, work absenteeism was in the range of 3%–8% in the United States [65]. The economic impact was small, with the pandemic reducing industrial production by about 1.2% in Canada during the peak and reducing GDP in the United States by about 1% [61]. Economic recovery following tapering off of the pandemic was almost immediate. Meanwhile, though hospital admissions in North America did increase during the pandemic, hospitals were able to accommodate surges in patient demand through repurposing beds, reassigning physicians, and cancelling elective surgeries [61]. This was complemented by a concerted effort to promote homecare for uncomplicated cases [61]. In the United Kingdom, these efforts were compromised by a regulation that individuals required a signed doctor’s note to qualify for sickness benefit [64].

The Asian flu was the first pandemic to occur in an environment with the global surveillance systems and laboratory capabilities in place to study it. In 1957, a worldwide network of laboratories was linked to the Influenza Research Center based in London, and investigators from Melbourne to Washington were able to study the strain soon after it emerged [64]. This was the first time that comprehensive surveillance was used to track the spread and burden of the disease, although expertise and methodological rigour were still lacking in this area [51]. It was also the first chance to study the response of an immunologically naïve population to influenza vaccination campaigns [45]. Development and distribution of vaccines was slow, and they were in only limited circulation by August 1957 in North America, and by October in Europe [64]. Vaccine allocation in the United States was based upon population size, with priority given to high risk individuals and essential personnel, an allocation strategy commonly employed today [61]. Prioritization was important, for by the end of the pandemic, only thirty million vaccine doses (enough to cover 17% of the American population at the time) had been distributed globally [66]. Despite a heavy focus on vaccination campaigns and a vaccine efficacy of 53%–60%, inadequate coverage prevented vaccination from having a significant impact on pandemic trends [61,66]. Antivirals had yet to be developed. Physicians took variable approaches to treatment with antibiotics, with some prescribing antibiotics to all cases and others only to the seriously ill [64]. While this might have helped reduce the burden of secondary bacterial infections, antibiotics are not effective against viral infection, and should not have been used to treat uncomplicated influenza. There was little use of non-pharmaceutical interventions, such as school closure, travel restrictions, banning of mass gatherings, or quarantine [65]. Quarantine, in particular, was considered inappropriate due to the mild nature of symptoms and the large overall number of infections. Though a mild influenza pandemic, the Asian flu provided a reminder of the persisting threat of the global spread of emergent diseases.

## 7. 1968–1970: Hong Kong Flu

It would be only a decade before the next global influenza pandemic, during which time there were few developments of note in terms of either medical science or globalization. This inter-pandemic period from 1959–1968 is not further addressed here, as it represents more of a continuation and expansion of past developments than a shift in how influenza was understood or addressed. One exception of note is that the Hong Kong flu was the first virus to exhibit an accelerated spread due to extensive air travel [67,68].

Ten years after its appearance, the Asian flu strain underwent an antigenic shift, reassorting to H3N2 and emerging as a new pandemic known as the Hong Kong flu. Despite being highly transmissible, this strain was even milder than the Asian flu. Estimates suggest that Spanish flu resulted in global excess all-cause mortality of 598 deaths per 100,000 people per year, whereas the same approximation for Asian flu was only 40.6; excess mortality during the Hong Kong flu was 16.9 [69]. However, methods for calculating these estimates varied across the three pandemics and may not be directly comparable. First reported in Hong Kong in July 1968, the virus spread was driven in part by Vietnam War veterans returning to the United States [67,70]. The infection was isolated in the United States and Japan in August; England, Wales, and Australia in September; Canada in December; and France in January 1969 [63]. 

Again displaying the characteristic shift in mortality towards younger populations, the highest case fatality rates were reported among children [71]. A unique feature of this pandemic was that the majority of influenza-related deaths in the United States (70%) and Canada (54%) occurred in the first pandemic wave, whereas countries in Europe and Asia experienced 70% of deaths associated with the pandemic during the second wave [63]. This was likely due to an antigenic drift between waves resulting in geographic variation in infection rates, but stands in contrast to the previously observed uniformity of a more severe second wave. The health burden was also two to five times smaller in North America than elsewhere [63].

Similar to the Asian flu, the Hong Kong flu is estimated to have caused between 500,000 and two million deaths worldwide [26,72]. Mortality rates were low, which may have been due to pre-existing immunity to the neuraminidase antigen (N2), the same as the previously circulating influenza strain. Still, during the two pandemic waves, the United States experienced a 47% increase in mortality related to pneumonia and influenza and a 6.6% increase in all-cause mortality; in Canada, these figures were somewhat lower at 43% and 3.6%, respectively [63]. However, the pandemic burden was higher in other countries, with increases in excess all-cause mortality of 9.1% (Australia), 11.9% (France), and 13.0% (England and Wales) over the previous year’s baseline [63]. These differences indicate the geographic heterogeneity of pandemic impact.

The social and economic burden of the Hong Kong flu was small, particularly in North America. There was some direct economic impact related to higher school and workplace absenteeism, but there was a rapid recovery after infection rates declined [61]. Low disease severity and mortality rates meant that more costly non-pharmaceutical interventions, such as school closures or quarantines, were unnecessary [73]. 

Instead, infection control measures emphasized a combination of vaccination, hospitalization for complicated cases, and antibiotics to treat secondary (bacterial) pneumonia. In most countries, vaccines were not available until after the pandemic had peaked [74]. Meanwhile, surges in hospitalizations caused problems in some areas, with an excess hospitalization rate of 150% reported in Portland Oregon, for 1968–1969, relative to 1970–1971 [75]. Hospitalization was significantly more likely among the elderly, and occurred at a rate that would be impossible to accommodate today [76]. This is because, in general, hospital bed capacities have either decreased or not increased sufficiently to keep pace with population growth rates [77]. The characteristics of the Hong Kong flu pandemic indicated a lack of progress in public health intervention strategies and medical science between the 1957 and 1968 pandemics.

## 8. 1970s–2000s: Computers, Drugs, and the WTO

Despite two global pandemics in a decade, popular opinion in 1970 was that societies had figured out how to prevent, treat, and control infectious diseases [78]. This may have been partly due to the less severe nature of the Asian and Hong Kong flus, which simplified responses and masked inefficiencies. Confidence was further fuelled by the success of vaccines in reducing morbidity and mortality from both smallpox and poliomyelitis, particularly in Western countries. In 1969, the United States Surgeon General declared it was time to ‘…close the book on infectious diseases, declare the war on pestilence won’ [79]. In time, this optimism would be challenged by outbreaks of various infectious diseases, including influenza, HIV/AIDS, and SARS. In fact, Jones and colleagues reported the emergence or re-emergence of several hundred new infectious diseases between 1940 and 2004, most of which have appeared since 1970 [80]. This points to a trend of increasing exposure to emerging infectious diseases, driven by globalization processes, that has been balanced, to some degree, by an increasing capacity to control infectious disease outbreaks, driven by advances in public health advances. 

In 1977, H1N1 re-emerged for the first time since before the 1957 pandemic to cause a “pseudo-pandemic” known as the Russian flu, which spread through the Soviet Union, Hong Kong, and China [2]. Disproportionately affecting those born after 1957, but not resulting in a significant increase in illness and death, it did not spread widely enough to produce a pandemic [13,73,76]. Though it is not clear how H1N1 re-emerged, it is suspected to have arisen from a laboratory accident [2]. Notably, this was the first time that two distinct influenza strains produced persistent co-circulation, as neither H1N1 nor H3N2 was displaced [76]. Both remain dominant sources of global influenza infection today. Later on, 1997 would mark the first case of human infection with H5N1 avian influenza, sparking fears of another pandemic from a pathogenic influenza strain [13]. However, in the twenty years since its emergence, H5N1 influenza has failed to achieve substantial human-to-human transmission. This may be because the virus attaches to, and replicates in, cells of the lower respiratory tract, in contrast to most influenza strains which attach and replicate in the upper respiratory tract [13]. 

An important development in this time was the growing availability and functionality of computer technology. Though the first digital computer was built in 1946, it was not until 1969 that host computers would be connected on a common network, and not until 1991 that the World Wide Web was introduced [81]. In addition to allowing the expanded use of sophisticated predictive modeling programs, the rise of computers and the Internet also had significant implications for surveillance capabilities. The WHO had established a Global Influenza Surveillance Network in 1952 (renamed the Global Influenza Surveillance and Response System in 2011) to facilitate monitoring of influenza infections throughout the world. It was not until the establishment of FluNet in 1996, however, that surveillance information became publicly available in near-real-time. Today, FluNet is made up of 138 National Influenza Centers, six WHO Collaborating Centres, and four Essential Regulatory Laboratories [82]. This network monitors the influenza strains circulating in human populations, speeding up vaccine development and stockpiling, and acts as a warning system to inform other preparedness activities. Meanwhile, federal and regional health agencies scaled up surveillance practices [83,84]. However, most surveillance is based solely upon patients who seek medical care, thereby excluding asymptomatic or self-limiting infections, underestimating the burden of influenza, and missing important routes of disease transmission [83]. Moreover, parallel expansion of federal and regional health agencies was not accompanied by stringent protocols to facilitate inter-agency cooperation. This has led to a problematic lack of coordination in response to recent infectious disease outbreaks, such as the reaction to the 2002–2003 SARS outbreak in Canada [50].

During the late twentieth century, there were two particularly important medical advances: the purification of vaccines and the development of antivirals to treat influenza. Previously, impurities in influenza vaccines would sometimes cause flu-like symptoms, with a risk of more serious complications. In 1976, for example, influenza vaccination in the United States was linked with an increased risk of Guillain-Barré syndrome, a serious, though poorly understood, neurological condition that can lead to paralysis and death [2]. From forty million vaccinations, there were 532 cases and thirty-two deaths reported [85]. Side effects in more recent vaccines tend to be mild, with no significant increase in risk of Guillain-Barré syndrome having been reported since the 1976 vaccine [86]. Meanwhile, antiviral drugs were developed to combat influenza infection. In 1964, amantadine was reported as an inhibitor of influenza, and was particularly useful for prophylaxis (infection prevention) [87]. Though this class of drug is susceptible to viral development of drug resistance, it also provided a basis for the more recent development of neuraminidase inhibitors (generic names zanamivir and oseltamivir), which can be effective in preventing influenza infection, as well as reducing the severity and duration of infection, particularly if administered within 48 hours of symptom onset [88]. Neuraminidase inhibitors are the class of antiviral most commonly used today: their development was an important milestone in increasing response capabilities to influenza.

Advances in technology and international economic cooperation dramatically increased the movement of people and goods across borders. The Boeing 747, the first “wide-body” passenger jet, was invented in 1969, and air travel became increasingly popular in the 1970s as its cost decreased [81,89]. In the United States, for example, the number of annual foreign visitors grew a hundred-fold by the turn of the century [54]. By 2004, the number of people who crossed international borders that year (743 million) was 73% higher than in 1989 [90]. At the same time, the 1995 foundation of the World Trade Organization, a successor to GATT, heightened the pace of multilateral international trade [55,89]. Over the following years the value of merchandise exports and commercial services trade increased by an average of 7.3% and 8.2% per year, respectively [55]. By the time swine flu emerged in 2009, global connectedness was of an entirely different scale than during previous pandemics. This would have important implications for disease emergence, spread, impact, and surveillance. 

## 9. 2009–2010: Swine Flu

The pH1N1/09 virus, also known as swine flu, Mexican flu, New flu, and A(H1N1), likely emerged from Mexico in April 2009 [91,92]. It was first recorded in almost simultaneous outbreaks in Mexico and the United States [26]. Within weeks, the disease had spread across 30 countries [93]. On 11 June 2009, the WHO declared a global influenza pandemic [94]. The extent of global trade and travel allowed swine flu to spread as widely in six weeks as past pandemics had in six months. By July, infection was reported in 122 countries, with 134,000 laboratory-confirmed cases and 800 deaths [61]. In this case, the pandemic emerged as a result of triple viral reassortment between two influenza lineages that had been circulating in pigs for years [93]. The viral genes appear to have come from viruses found in North American and Eurasian swine, though it is unclear when and where reassortment occurred [26]. 

As with the pandemics of the twentieth century, the swine flu pandemic exhibited wave behaviour, with wave timing varying geographically. In Mexico, for example, a three-wave pandemic profile has been identified, with a spring, summer, and fall wave [95]. Pandemic peaks in the rest of North America were more consistent with a two-wave profile, with a spring-summer (29 March–2 August 2009) and fall (2 August–31 December 2009) wave [96]. Elsewhere, the timing of pandemic waves was very different. In Europe, despite heterogeneity in pandemic timing, the general pattern was of an initial, generally mild wave in the spring and early summer of 2009, which subsided as the summer progressed, only to re-emerge with the re-opening of schools to produce a more severe second wave [97,98]. India, meanwhile, experienced three wave peaks in September 2009, December 2009, and August 2010 [99]. The WHO declared the pandemic officially over in August 2010 [97]. After the pandemic, there were 18,500 laboratory-confirmed deaths globally, though mathematical models suggest that actual influenza-associated mortality was somewhere between 151,700 and 575,400, eight to thirty times the number confirmed in laboratory [100,101]. It should be noted that the mortality estimates for twentieth century pandemics did not rely on laboratory-confirmed cases, but rather on statistical attribution of excess all-cause mortality [102]. The latter method is more inclusive, as the former risks influenza-associated deaths being under-reported, due to misattribution of cause of death or the absence of laboratory confirmation of infection. As a result, the health burden of pH1N1, relative to past pandemics, may have been underestimated. 

Again, pandemic infection resulted in a shift in mortality towards younger populations, primarily affecting children, young adults, and pregnant women [103], with thirty-seven years being calculated as the average age for laboratory-confirmed deaths in the United States [102]. As a result, years of life lost were again disproportionately higher for the pandemic strain than seasonal influenza. It was estimated that, between May and December 2009, pH1N1-attributable illness resulted in between 334,000 and 1,973,000 years of life lost, figures that are comparable to a typical seasonal flu at the lower bound and to the 1968 pandemic at the upper bound [102]. 

The pandemic also caused societal disruption and a substantial economic burden, which was documented more comprehensively than for past influenza pandemics. However, the total global impact of the pandemic is not well understood [103]. First, direct costs related to treatment, with respect to drugs, outpatient visits, and hospitalizations, were high. In Canada, total costs have been estimated at around CAD$2 billion, with the care of hospitalized patients alone estimated to be close to CAD$200 million, as the cost of hospitalization for each H1N1-infected patient averaged about $11,000 [104]. Emergency department visits are estimated to have resulted in costs of another $50 million [104].

Overall, estimates of economic losses range from 0.5%–1.5% of GDP in affected countries [105,106]. Such calculations, however, tend to underestimate other, often longer-lasting impacts related infection prevention efforts, such as school closures, lost productivity from work absenteeism, shifts in consumer habits, and reduced tourism. For example, although reactive school closures were implemented in many countries due to the high transmission rate in children [106], associated costs are difficult to calculate, as such action also leads to work absenteeism and lost productivity [107], One study of the impact of school closure on households in New York City found that, in 17% of households, at least one adult had to miss work because of the closures [108]. Though estimates vary depending on the size of the affected population and duration of closure, school closures have been estimated to cost from tens to hundreds of millions of dollars [109]. The pandemic also negatively affected global tourism [110], with airlines reporting losses in the tens of millions [105]. It is difficult, however, to disentangle swine flu’s role in this decline, as the global economic crisis of 2008 was occurring simultaneously.

The response to the 2009 H1N1 pandemic, particularly in North America and Europe, demonstrated a significantly improved level of preparedness relative to past pandemics. This was the result of emergency preparedness efforts catalyzed by the earlier SARS outbreak of 2002–2003 and persisting fears surrounding H5N1 avian flu. Containment efforts employed a combination of pharmaceutical and non-pharmaceutical interventions. In the United Kingdom, for example, an aggressive containment campaign combined school closure and voluntary isolation with antiviral treatment for suspected cases and mass prophylaxis of potential contacts; these interventions helped control the outbreak until more information could be gathered [111]. The swine flu pandemic also marked the first pandemic response combining both vaccination and antiviral use. In Canada, though an H1N1 vaccine was not approved until about six weeks into the second wave, the largest mass immunization program in the nation’s history was carried out, with the federal government investing $400 million to purchase fifty million doses of the vaccine [112]. High priority groups were the first to receive vaccination, before it was expanded to all groups a few weeks later [113]. Between one third and one half of the population was vaccinated over the remainder of the pandemic [114]. Vaccination coverage was lower in the United States (with state averages from 12.9% to 38.8%) [115], and much of Europe, with the exception of Norway (45%) and Sweden (59%) [116]. Unfortunately, there was little use of antivirals before September 2009, though awareness campaigns targeting primary care providers increased their use to treat patients later in the pandemic [104].

Non-pharmaceutical measures applied in response to past pandemics were again widely implemented to help contain the pandemic. The most common among these were recommendations for hand hygiene and voluntary isolation of symptomatic individuals [117]. Canada did not recommend school closures to mitigate the pandemic [118], but did benefit from closures for summer break during the first wave; estimates from Alberta suggest this reduced transmission among children by at least 50% [119]. Other countries, including the United States, United Kingdom, and Australia, did recommend and implement school closures [108,118,120]. While there is uncertainty regarding the effectiveness of these interventions, research suggests strong compliance with, and public acceptance of, these measures [120].

While understanding of the influenza virus’s properties and transmission was by this point fairly advanced, weaknesses in maintaining consistent diagnostic protocols presented challenges for surveillance efforts [104]. Officials tracking data on hospitalization, intensive care admission, and mortality struggled to generate relevant surveillance data to inform decisions in real-time [121]. Instead, summary data was made available after the fact. The Public Health Agency of Canada (PHAC) surveillance group, for example, consisted of only four people at the time of pandemic onset, making it difficult to produce, analyze, appraise, and communicate relevant data in a timely fashion [121]. This was compounded by shortcomings in inter-agency coordination, highlighting a dual need for clarification of roles and responsibilities in planning and response, and for a streamlined approach for the incorporation of evidence into decision-making processes [118].

Another important concern was the observed strain on public health, hospital, and human resources during pandemic peaks [104]. While health systems were generally able to accommodate surges in patient demand, it is likely that an even marginally more severe pandemic would have resulted in harmful service disruption and the need to turn patients away [121]. This was, in part, due to the need for doctors to issue antiviral prescriptions, which has since been addressed by extending this authority to pharmacists. Overall, the 2009 pH1N1 pandemic was a mild, albeit costly, global virus. While it has reinforced optimism about pandemic preparedness, it should not necessarily be seen as predictive of future pandemic severity.

## 10. 2010–2016 and Beyond

Since the 2009 pandemic, H1N1 and H3N2 have continued to circulate in the global population. However, the novel pandemic strain pH1N1/09 displaced the previously circulating H1N1 strain to begin producing seasonal outbreaks [122]. There have been reports of human infection with avian strains for which humans are immunologically naïve, particularly H5N1 and H7N9, but these infections have failed to achieve human-to-human transmission [123,124]. Despite expansion of surveillance efforts, it is impossible to predict with certainty whether the next pandemic will arise from an antigenic shift in currently-circulating strains or a mutation that enables human-to-human transmission in one of the more lethal avian strains. Animal husbandry continues to increase contact between humans and animals, providing opportunities for both viral mixing between animal hosts and spillover to human populations. There have been massive increases in domestic animal populations, with poultry and swine being of particular relevance to influenza risk. Keeping animals at high population densities facilitates viral reassortment through shared habitats and drinking water, as animal influenza is passed via the oral-faecal route. There have recently been more frequent influenza outbreaks among domestic poultry populations [125,126], and a greater diversity of influenza viruses circulating among pig populations [126,127], both increasing the risk of another human pandemic. Most human infections with animal influenza arise from close, direct contact with poultry or swine [128]. Live poultry markets in particular have been identified as a major source of viral mixing, as well as human H5N1 infection [123,129]. This suggests a need for international discussion and cooperation towards policy development to reduce live market practices and mitigate associated risks.

The year 2014 marked the first time that flights per day exceeded an annual average of 100,000, while 2013 was the first time that annual passengers exceeded three billion [130]. Meanwhile, global population growth continues. When the 1918 pandemic occurred, the global population was around 1.8 billion [52]; as of July 2016, the World Population Clock estimates a global population of about 7.4 billion [131]. If a pandemic today were to kill the same proportion as in 1918, this would equal between 74 and 370 million people. Population growth, human mobility, and greater proximity to animal reservoirs continue to increase both the risk of pandemic emergence and the speed with which such a pandemic could spread across the globe. Between 1700 and 1889, the average inter-pandemic period ranged from 50–60 years; since 1889 this period has shortened to 10–40 years [12]. While a pandemic one hundred years ago would take weeks or months to spread globally, an infection today could spread to every continent in days. This increased risk can only be addressed by a combination of local, national, and international efforts to improve both mitigation and containment of future pandemics. 

There is also a need for improved global surveillance capabilities of both humans and animals. A weakness noted in the aftermath of the swine flu pandemic was the need for stronger surveillance of swine populations [93,121], with the same need holding for avian reservoirs. While recent efforts have yielded some results, more investment is needed to scale up animal surveillance in areas where viral spillover from animal to human populations is most likely to occur. Meanwhile, governments must increase global cooperation to advance early warning systems. This is because contact tracing for influenza is difficult and expensive, even in an outbreak involving a very small population [132]. As such, less developed countries may lack the resources to carry out consistent and sufficient disease surveillance; it becomes very challenging to trace contacts once the disease has spread internationally. An important effort will be to streamline the disease reporting process, shortening lag time between physician reporting and national and international evaluation. The fact that disease surveillance relies on hospital, outpatient, and physician visit reporting means that outbreaks are reported, at the earliest, when an individual seeks care. In the case of influenza, this may be sufficiently rare to prevent detection before substantial community spread has occurred. It also ignores findings that a substantial health and economic burden associated with pandemic influenza arises from the morbidity from uncomplicated, self-limiting cases for which individuals do not seek treatment. As such, population-based surveillance should be strengthened, exploiting social media capabilities to obtain real-time data.

In short, while medical advances and past experience with less severe pandemics may lead some to a degree of complacency, globalization processes have increased the risk of emergence and spread of a novel influenza strains. Since the characteristics of such outbreaks are difficult to predict in advance, global capabilities must continue moving towards information provision in real-time, with data that can be streamlined into flexible models to inform policies and programs as the situation develops. In addition to enhanced surveillance capabilities, this effort requires expanded availability of quick, affordable diagnostic tests, and a consistent approach to diagnostic reporting.

## 11. Conclusions

This review has examined the ways in which the understanding, experience, and response to pandemic influenza has evolved over time. While significant progress in reducing pandemic impacts has been made, thanks in large part to advances in pharmaceutical interventions and surveillance, there is much about pandemic influenza that is still poorly understood. The emergence of pandemics has not adhered to typical influenza seasons, and can occur at any time. Meanwhile, though pandemics tend to occur in waves, it is difficult to predict why, how, and when waves will occur in different countries. Increased human preparedness has been accompanied by an increased exposure to, and frequency of, pandemic spillover from animal to human populations. Pandemic transmission has always occurred along the dominant lines of movement and communication at the time. In the past, pandemics spread somewhat predictably along military passages or important trade routes, but globalization has multiplied and obscured the dominant routes. Given their sheer number, and the speed with which human and animal transmission vectors can move, there is no longer a single dominant pathway for the geographical movement and expansion of infectious diseases. Pandemics are inherently uncertain, necessitating policies that are flexible in responding to outbreaks as they develop. While insights can be gathered from past experiences of pandemic influenza, it is unlikely that the next event will mimic those of the past. Continued efforts are required to improve local, national, and international surveillance, coordination, and resource planning to most effectively mitigate and contain future pandemics. Despite all of the uncertainty surrounding pandemics, history has shown that influenza pandemics occur in cycles, albeit unpredictable ones, and that it is not a question of whether another influenza pandemic will occur, but when. 

## Figures and Tables

**Table 1:** Summary of the key characteristics of influenza pandemics from the past one hundred years.

Pandemic Name	Year	Strain	Suspected Origin of Outbreak	Approximate Number of Deaths
Spanish flu	1918–1920	H1N1	China	40–50 million
Asian flu	1957–1958	H2N2	China	1–2 million
Hong Kong flu	1968–1970	H3N2	China	500,000–2 million
Swine flu	2009–2010	H1N1	Mexico	Up to 575,000
